# Exploring purchase intentions of new energy vehicles: Do “mianzi” and green peer influence matter?

**DOI:** 10.3389/fpsyg.2022.951132

**Published:** 2022-09-13

**Authors:** Haibo Zhao, Rubing Bai, Ran Liu, Hong Wang

**Affiliations:** ^1^School of Management and Economics, Beijing Institute of Technology, Beijing, China; ^2^School of Economics and Management, Beijing University of Agriculture, Beijing, China

**Keywords:** new energy vehicles, purchase intention, norm activation model, mianzi (face), green peer influence, green self-identity

## Abstract

New energy vehicle is an innovative means of transportation, and its development has been widely concerned all over the world. However, few studies investigate the purchase intention of new energy vehicles (NEVs) from the perspective of combining altruism and cultural factors. Based on the extended norm activation model (NAM), this study explores the influencing factors of NEVs’ purchasing intention and the moderating effects of “mianzi” and green peer influence. According to 302 valid questionnaires, the results indicated that the extended NAM model is useful in predicting consumer purchasing behavior with an improved explanatory power in purchase intentions of NEVs from 15 to 26%. The awareness of consequences, the ascription of responsibility, and green self-identity have a positive impact on the personal norm. Personal norm and green self-identity are positively associated with purchase intention. “Mianzi” and green peer influence positively moderate the relationship between green self-identity and intention to purchase. The findings give new insights into the impact of cultural factors on purchasing NEVs and profound suggestions for policymakers and enterprises to promote the development of NEVs.

## Introduction

With socioeconomic development and urbanization, energy crisis and environmental issues have become more prominent, which has attracted the common concern of all countries globally. Transportation industries cause severe ecological problems, such as greenhouse gas and haze (Zhang et al., 2015; Miao et al., 2019). The enormous growth of car ownership is a significant contributor to energy consumption and environmental issues (Wang and Dong, 2016). About 1.1 billion tons of CO_2_ is emitted into the atmosphere in China’s transport industry in 2018, making up approximately 11% of the country’s total energy-related CO_2_ emissions (Energy Foundation [EF], 2020). According to the International Energy Agency (2009), the transportation sector contributes to 23% of the total global carbon dioxide emissions, ranking as one of the primary sources of carbon dioxide emissions. New energy vehicles (NEVs) may play a significant role in efforts to decrease vehicle exhaust emissions and increase ecological sustainability (Linn and McConnell, 2019). The Chinese government has issued a set of measures to encourage the purchasing of NEVs, such as building charging facilities and providing considerable subsidies (Wang and Dong, 2016). Besides, the Chinese government has set a practical plan for more than 5 million NEVs by 2020 (Zhang X. et al., 2013). However, consumers’ purchasing power for NEVs is insufficient, and the market share remains low (He and He, 2015). For example, the sales volume for NEVs in 2015 was 331,000 in China, taking up approximately 1.34% of the total sales of cars, while the number of NEVs was only 580,000 at the beginning of 2016. In Norway, an early adopter of electric vehicles, pure electric cars account for only about 2% of all passenger vehicles (Bjerkan et al., 2016). Therefore, it is essential to investigate the factors that affect the purchase of NEVs, which is crucial to develop effective strategies to enhance the sales of NEVs for enterprises.

The existing research on the purchase intention of NEVs primarily focuses on cost factors (De Haan et al., 2006; Dong et al., 2020); government policy incentives (Chi et al., 2021; Liu et al., 2022), product attributes (Kang and Park, 2011; Su et al., 2020), and psychological factors (Huang and Ge, 2019; Xu et al., 2020). The theoretical frames of some studies were mainly based on the theory of planned behavior, that is, the technology acceptance model (Wang and Wang, 2013; Huang and Ge, 2019). In addition to cognitive factors and rational choice, cultural factors (Faiers et al., 2007) and moral factors can also influence sustainable consumption behavior. It is reasonable to infer that moral and cultural factors are as vital as rational and cognitive factors in understanding consumers’ purchasing behavior. Chinese consumers, guided by Confucian culture, may consider their social reputation and opinions during purchasing decisions (Guo and Lin, 2015). “Mianzi” is a dimension of Confucian values, profoundly embedded in Chinese market habits (Lao and Wang, 2015). China is a collectivist culture country, and collectivist societies exhibit high degrees of group conformance (Vignoles et al., 2006). Individuals seek conformance from the group, and individual’s sense of belonging to a particular group is a vital factor in consumer behavior. Peer influence is a significant predictor of consumers’ decision to buy green goods (Suki and Suki, 2019). Nevertheless, there are few studies in the literature that combine cultural factors and moral factors to analyze the intention to purchase NEVs, and cultural factors are rarely used as moderating variables. Based on the norm activation model, this study adds the cultural elements of “mianzi” and green peer influence as moderating factors, which explores their impact on the purchasing intention of NEVs to fill this research gap.

The rest of this article is arranged as follows. In section “Literature review,” we propose a literature review on NEVs and norm activation model (NAM). Section “Research model and hypotheses” introduces the research model and hypotheses. The research method is presented in section “Research method.”. We analyze the data and present the research findings in section “Data analysis and results.” In the last section, the research conclusions, research implications, and research limitations are presented.

## Literature review

### New energy vehicles

In the backdrop of the energy crisis and environmental damage, it is necessary to use sustainable transportation innovation to reduce carbon dioxide emissions. Compared to conventional vehicles, NEVs offer certain obvious competitive advantages, such as lower pollutant emissions, savings on non-renewable energy, and environmental friendliness (Hofmann et al., 2016). They are an essential means of improving the quality of our environment. Most previous studies adopt purchase intention instead of actual behavior, since purchase intention is regarded as the proxy of real action. Following the idea of prior literature, this study employs purchase intention to analyze the influencing factors of NEVs.

Recently, many researchers have studied the intention to purchase NEVs. Based on previous studies, the influencing factors can be concluded as three aspects: product attributes, consumer characteristics, and environmental factors. Product attributes involve the price of the vehicle, the cost, maximum cruising range, battery range, the technology of the car, and performance (Ozaki and Sevastyanova, 2011; Lopes et al., 2014; Chen et al., 2019; Dong et al., 2020; Su et al., 2020). Consumer characteristics mainly contain the demographic factors, personal traits, socioeconomic characteristics, and psychological factors of consumers, such as environmental awareness and environmental innovativeness (Kim et al., 2018; Huang and Ge, 2019; He et al., 2020; Wang et al., 2020; Wang et al., 2021; Lin and Shi, 2022). Environmental factors refer to tax incentives, national policy, government subsidies, and infrastructure (Tan et al., 2018; Li et al., 2020; Yu et al., 2020; Chi et al., 2021; Zhao et al., 2021; Qin and Xiong, 2022).

The majority of recent research has clarified the consumers’ purchase behavior of NEVs based on established theoretical models, for example, the theory of planned behavior, diffusion of innovation theory, norm activation model, technology acceptance model, induced innovation theory, grounded theory, and construal level theory (Peters et al., 2015; He and Zhan, 2018; Huang and Ge, 2019; Dong et al., 2020; Su et al., 2020; Liu et al., 2021; He et al., 2022; Lin and Shi, 2022). Most theoretical models assume that consumers make purchasing decisions in the light of their rational assessment and benefits, and explain consumers’ behavior to buy NEVs from the perspective of self-interest. However, the NEV is a kind of environmental protection vehicle, and its adoption has the attribute of both self-interest and altruism. Extant studies rarely explained the purchase behavior of NEVs according to the perspective of combining altruism and cultural factors. Thus, it is vital to study people’s purchasing behavior toward NEVs from an altruistic perspective. Meanwhile, socio-cultural factors are important variables affecting consumer’s choice behavior.

### Norm activation model

The norm activation model (Schwartz, 1977) is a critical theory to predict people’s behavior from altruism. The theory is widely used to explain energy conservation behavior (Wittenberg et al., 2018), sustainable transport behavior (He and Zhan, 2018), recycling behavior (Wang et al., 2018), household PM2.5-reduction behavior (Shi et al., 2017), and energy-saving behavior (Zhang Y. et al., 2013). NEVs are widely known as eco-sustainable transport systems. They alleviate serious environmental concerns by reducing energy consumption and harmful emissions. Meanwhile, it contributes to socially sustainable development and environmental protection. Hence, it is significant to study the influence mechanism of purchase intention for NEVs based on the norm activation model.

The norm activation model consists of four variables: awareness of consequences, the ascription of responsibility, personal norm, and behavior. The awareness of consequences and ascription of responsibility are predictor variables of the personal norm, and in turn, directly affect people’s behavioral intention or actual behavior. Different scholars have different views on the relationship between variables in the norm activation model. Some scholars hold that NAM is a mediator model (Steg et al., 2005), while other scholars consider NAM as a moderator model (Hopper and Nielsen, 1991). According to the statistics of previous studies, it is evident that NAM as a mediator model is widely used to investigate pro-environment behaviors. Therefore, we adopt the mediator model of the NAM in this study.

To increase the prediction ability of the model, scholars usually expand NAM in terms of two aspects: increasing the predictive variables of the personal norm and adding external factors. For example, He and Zhan (2018) built a NAM to analyze the intention of customers to purchase electric vehicles by incorporating perceived consumer effectiveness and external costs. Wu et al. (2022) suggested an expanded NAM that included face consciousness in evaluating the ecologically responsible behavior of Chinese visitors. China has a collectivist culture. People have a strong communal awareness and are inspired by Confucian culture. Mianzi is a part of Chinese culture. People are willing to adjust their actions to get social acceptance and to be consistent with the other group members. When making purchasing choices in China, consumers may consider their public image, as well as the views of others. They are more likely to purchase goods (e.g., green products) that can enhance their social image and reputation. Furthermore, it has been discovered that green self-identity is a significant predictor of customer intention to purchase green goods. Based on the self-congruity theory (Sirgy, 1986), individuals who identify as green customers may choose eco-friendly goods to meet their self-definition demands and obtain personal satisfaction. Therefore, combined with cultural factors, the research expands the NAM by introducing three variables to analyze the purchasing intention of NEVs: green self-identity, “mianzi,” and green peer influence.

## Research model and hypotheses

**FIGURE 1:**
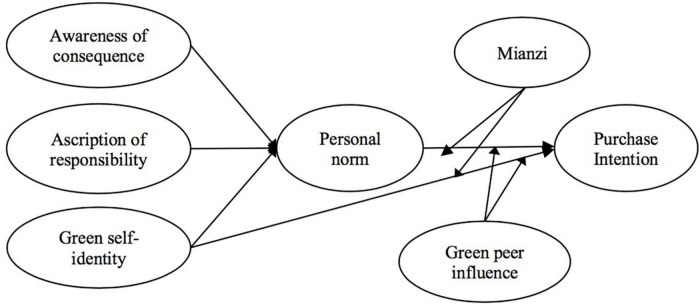
Research model.

As shown in Figure 1, we propose the research model for this article. In the research model, green self-identity, awareness of consequences, and ascription of responsibility are predictors of personal norm. Green self-identity and personal norm are predictive variables of purchase intention of NEVs. Furthermore, green peer influence and “mianzi” moderate the effects of green self-identity and personal norm on purchase intention.

Based on norm activation model, awareness of consequences and ascription of responsibility are positively correlated with personal norm (Schwartz, 1977). Awareness of consequences refers to the individual’s consciousness of the adverse consequences that the individual does not carry out ecological behavior. Ascription of responsibility refers to the individual’s feeling of responsibility for detrimental outcomes caused by not practicing environmental behavior (De Groot and Steg, 2009). Facing severe environmental problems, if consumers realize that conventional vehicles lead to environmental pollution and make the environment worse, their sense of ecological moral responsibility will be motivated and tend to perform pro-environmental behaviors (Harland et al., 2007). Furthermore, if people consciously realize the negative environmental impacts caused by conventional vehicles, they will feel responsible for the negative consequences and have a moral responsibility to choose NEVs to fulfill the obligation of environmental protection (Han, 2014). In summary, we propose the following hypotheses:

*H1. Awareness of consequences is positively related to consumers’ personal norm.*

*H2. Ascription of responsibility is positively related to consumers’ personal norm.*

Personal norm refers to the individual’s internal responsibility for the moral obligation of pro-social behavior (Schwartz, 1973). In the light of the norm activation model, personal norm is the predictive variable of actual action. People are more willing to perform environmentally friendly behavior to show their moral responsibility when personal norms are motivated (De Groot and Steg, 2009). Prior studies have confirmed a favorable association between personal norm and pro-environmental behavior. For example, Nordlund and Garvill (2003) indicated that personal norm is positively related to consumers’ environmentally friendly way of tourism. If consumers have set personal norms, they will easily recognize that traditional automobiles can pollute the atmosphere and have a moral obligation to take part in environmental conservation initiatives, thus motivating them to be more likely to buy NEVs. Accordingly, we hypothesize as follows:

*H3. Personal norm is positively correlated with consumers’ purchase intention of NEVs.*

Green self-identity refers to the extent to which an individual sees himself/herself as an environmentally friendly person (van der Werff et al., 2013; Nguyen et al., 2016). People who think of themselves as green consumers may have a sense of pride and a positive attitude toward environmental protection behavior. They tend to buy environmentally friendly products because eco-friendly products give them a sense of green identity and self-definitional needs (Bhattacharya and Sen, 2003). Meanwhile, existing studies have shown that green self-identity has a strong link with the personal norm. For instance, van der Werff et al. (2013) discovered that green self-identity is strongly associated with moral obligation when purchasing green energy goods. Camilla et al. (2017) considered that green self-identity affects the intention to adopt electric cars. That is to say, consumers with strong green self-identity are more likely to have the ethical obligation to be engaged in environmentally sustainable actions. Given the above analysis, we propose the following hypothesis:

*H4. Green self-identity positively affects consumers’ personal norm.*

Previous findings have shown that the association between green self-identity and environmentally sustainable behavior is strong. For instance, Barbarossa and De Pelsmacker (2016) suggested that green self-identity has a strong connection to the purchasing intention of ecologically sustainable goods. Meanwhile, Nguyen et al. (2016) observed a favorable association between green self-identity and pro-environmental purchase behavior. In other words, consumers with a high level of green self-identity appear to be more concerned about the environment. Thus, the following research hypothesis was established:

*H5. Green self-identity positively affects consumers’ purchase intention of NEVs.*

As a collectivist country, Chinese culture advocates Confucian. “Mianzi” is an important aspect of Confucian values, which affects consumer behavior and decision-making. “Mianzi” means individual public image in society. The idea of “mianzi” is profoundly ingrained in the consumption concept of Chinese consumers (Lao and Wang, 2015) and represents the status of consumers, and is also considered a key to explain the behavior of many Chinese people (Jiang, 2009). In purchasing decisions, Chinese consumers consider their social status of purchasing and refer to other people’s opinions (Guo and Lin, 2015). Consumers who place a high value on mianzi may pay particular attention to their public image by displaying a green image that complies with social values. Green products can not only represent the attitude of consumers, but also bring consumers a sense of identity. NEVs are typical green products and are high-involvement products. NEVs are products with environmental protection image, and can give people a sense of “mianzi.” Meanwhile, “mianzi” makes consumers vulnerable to social norms, and they are more sensitive to the signal effect of green consumption. They obtain psychological benefits from their green consumption and publicly show a pro-environmental image. Consumers’ pro-social considerations of image and social status can drive consumers to buy NEVs (Graham-Rowe et al., 2012). Therefore, we hold that “mianzi” can strengthen people’s green identity and moral responsibility in the process of purchasing NEVs in the context of Chinese society.

In addition, Wang (2013) revealed that “mianzi” positively impacts resource-saving consumption behavior. However, Chen et al. (2019) noted that the concept of “mianzi” is not positively associated with NEVs’ purchasing intentions. Hence, the research attempts to consider “mianzi” as a moderator variable. Therefore, we present the following hypotheses:

*H6a. The effect of personal norm on purchase intention of NEVs is positively moderated by “mianzi.”*

*H6b. The effect of green self-identity on purchase intention of new energy vehicles is positively moderated by “mianzi.”*

Green peer influence is referred to as the influence of social groups on individuals’ green purchase behavior (Arpita and Shivendra, 2017). Belonging to external influencing factors, it represents external pressure, which comes from classmates, colleagues, friends, and family. Individuals express their allegiance to organizations that exert stress on members to conform to social norms. Peer influence is shown to have a significant role in influencing customers’ decisions to buy environmentally friendly goods because peers may persuade others to understand the environmental benefits of goods and the severity of ecological issues (Tsarenko et al., 2013). Peer recognition of green products makes individuals tend to be consistent within their entire group and change their purchasing behavior to accept green products. Moreover, social identity theory emphasizes a person’s sense of oneness or belonging to a particular group (Bartels and Reinders, 2010). This also shows that individual consumption behavior will be affected by social groups. Therefore, it can be inferred that a statistically significant relationship exists between peer influence and green consumption behavior.

Previous studies rarely test the moderating effect of peer influence on consumers’ green purchase behavior. Most scholars hold that green peer influence is positively related to individual consumption behavior. For example, Lee (2008) asserted that green peer influence positively connects with green purchase behavior. Nekmahmud et al. (2022) discovered that subjective norms show a substantial positive link with green purchase intentions for European and non-European tourist groups. But some scholars hold different views. Shi et al. (2017) reported that subjective norm has no positive connection with PM2.5-reduction intention. Yadav and Pathak (2016a) deemed that there is no positive connection between subjective norm and the consumer’s choice to buy organic foods. In the light of the above analysis, the article attempts to regard green peer influence as a moderator variable and develops the following hypotheses:

*H7a. The effect of personal norm on purchase intention of NEVs is positively moderated by green peer influence.*

*H7b. The effect of green self-identity on purchase intention of NEVs is positively moderated by green peer influence.*

## Research method

### Measurement development

Based on previous studies on NEVs (Wang et al., 2021; Lin and Shi, 2022), this study adopts the questionnaire survey method to collect data. This study adopts mature measures from prior studies to ensure validity. The formal questionnaire consists of two parts: measurement items of variables and individual demographic information. The scale of awareness of consequences was derived from Zhang Y. et al. (2013) and He and Zhan (2018). The scale of the ascription of responsibility was derived from the study of Zhang Y. et al. (2013) and He and Zhan (2018). The scale of the personal norm was adapted from Smith and McSweeney (2007) and Shi et al. (2017). The measurement of green purchase intention was adapted from a prior study (Yadav and Pathak, 2016b). Items for green self-identity were developed according to Kelly et al. (2008) and Camilla et al. (2017). Items for green peer influence were derived from Lee (2009) and Arpita and Shivendra (2017). The scale of “mianzi” was adapted from previous works (Zhang et al., 2011; Chen et al., 2019).

The original items were in English. The study adopted a back-translation method to translate English measures into Chinese. Then, we invited three non-marketing students to review the questionnaire. According to their suggestions, the questionnaire was further revised. The final version of the questionnaire is more understandable from the respondent’s perspective. The study adopted a 7-point Likert-type scale to assess all the factors, where 1 strongly disagree and 7 strongly agree.

### Data collection

The questionnaire survey was distributed through Sojump.^1^ We put the questionnaire hyperlink in the WeChat group. The respondents can receive a monetary reward (a red envelope by WeChat) for completing the questionnaire. After deleting invalid questionnaires, a total of 302 valid questionnaires were obtained. Table 1 displays comprehensive sample data profiles. About 44.7% of the respondents were male, and this proportion is slightly lower than that of the female respondents. Respondents between 21 and 40 years old took up the majority, which accounted for 88.4%. For personal education, most participants held bachelor’s degrees or above and were students and staff. Moreover, a majority of participants earned more than 3,000 CNY a month.

**TABLE 1:** Demographic characteristics of the sample.

Variable	Categories	Frequency	Percent
Gender	Male	135	44.7
	Female	167	55.3
Age	≤20	11	3.6
	21–30	199	65.9
	31–40	68	22.5
	41–50	20	6.6
	≥51	4	1.3
Education	High school or below	6	2
	Junior college degree	19	6.3
	Bachelor’s degree	107	35.4
	Master’s degree or above	170	56.3
Occupation	Student	120	39.7
	Working	138	45.7
	Others	44	14.6
Monthly income (CNY)	≤3,000	103	34.1
	3,001–5,000	53	17.5
	5,001–8,000	43	14.2
	8,001–10,000	41	13.6
	>10,000	62	20.5

## Data analysis and results

This study adopted SPSS 18 and AMOS 20 to analyze survey data. First, the reliability and validity of the measurement model were examined. Second, all hypotheses were tested in this study.

### Measurement model

The study employed confirmatory factor analysis (CFA) to assess the reliability and validity of the measurement models before conducting the hypothesis test. As shown in Table 2, Cronbach’s alpha of each construct was greater than 0.7, and the composite reliability of each construct was above 0.7, with high internal consistency and reliable scales (Fornell and Larcker, 1981). Moreover, the study examined convergent validity. It can be observed that the scores of the average variance extracted (AVE) for variables ranged from 0.556 to 0.762 and exceeded the threshold value of 0.5. Therefore, the questionnaire obtained enough convergent validity (Bagozzi and Yi, 1988).

**TABLE 2:** Results of measurement model analysis.

Construct	α	CR	AVE
Awareness of consequence	0.828	0.832	0.556
Ascription of responsibility	0.864	0.873	0.699
Personal norm	0.841	0.853	0.660
Green self-identity	0.748	0.790	0.564
Mianzi	0.879	0.882	0.716
Green peer influence	0.927	0.928	0.762
Purchase intention	0.895	0.898	0.747

α, Cronbach’s alpha; CR, composite reliability; AVE, average variance extracted.

To ensure the discriminant validity, this study analyzed the correlation coefficient and the square root of the AVE of each construct. It displays the description of discriminant validity, as seen in Table 3. We can find that the square root of the AVE of each construct exceeded their corresponding correlation coefficients with the construct. Thus, the scales achieved sufficient discriminant validity (Paulraj et al., 2008).

**TABLE 3:** Discriminant validity analysis.

Constructs	1	2	3	4	5	6	7
Awareness of consequence	0.746						
Ascription of responsibility	0.074	0.836					
Personal norm	0.223	0.16	0.812				
Green self-identity	0.195	0.067	0.25	0.751			
Mianzi	0.156	−0.031	0.181	0.284	0.846		
Green peer influence	0.135	0.040	0.388	0.508	0.488	0.873	
Purchase intention	0.30	0.018	0.390	0.430	0.444	0.486	0.864

1. Off-diagonal elements are correlations between constructs; 2. Diagonal elements are the square root of average variance extracted.

This research gathered data from self-reports, and this approach may contribute to common method bias. We employed the standard assessment method that Harman’s single-factor test was implemented to evaluate the bias (Podsakoff et al., 2003). According to the findings of the analysis, all the items may be divided into seven independent factors, and the largest explained variance was 29.53%, which was less than the threshold value of 30%. Accordingly, it can be inferred that common method bias did not occur in the current study (Malhotra et al., 2006).

### Hypothesis tests

#### Results of path coefficient test

The original NAM model was analyzed, and the overall fit was considered to meet the recommended requirements (χ^2^/df = 1.595; CFI = 0.982; TLI = 0.976; RMSEA = 0.044; CFI = 0.954). Consequently, to increase the explanatory power of the original NAM model, one additional variable (Green self-identity) was included. The fit of the extended model was acceptable (χ^2^/df = 1.571; CFI = 0.976; TLI = 0.970; RMSEA = 0.044; CFI = 0.943). The results showed that adding an additional variable to the original NAM model increased the variance in NEV purchase behavior. The explanatory power increased from 0.15 to 0.26 in the final model.

**FIGURE 2:**
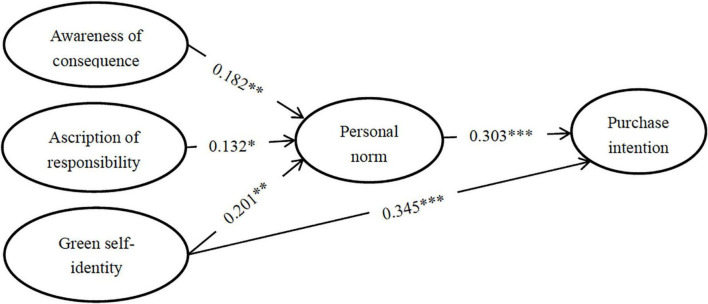
Results of hypothesis testing. *p < 0.05; ^**^p < 0.01; ^***^p < 0.001.

To calculate the path coefficient, the structural equation model was used in this study. The results of path coefficients are displayed in Figure 2. We can find that awareness of consequences is positively correlated to the personal norm (β = 0.182, *p* < 0.01). As such, H1 is supported. The path coefficient from the ascription of responsibility to the personal norm (β = 0.132, *p* < 0.05) is positive and significant. Hence, H2 is supported. Personal norm is a positive connection to purchasing NEVs (β = 0.303, *p* < 0.001). Therefore, H3 is supported. In addition, green self-identity is significantly linked to personal norm (β = 0.201, *p* < 0.01) and purchase intention (β = 0.345, *p* < 0.001). Hence, these findings support H4 and H5.

#### Moderating effect test

This study employed hierarchical regression analysis to examine the moderating effects. We conducted three procedures for moderating effects. First, to eliminate the influence of different measurements of variables on the statistical results, we standardized the independent variable and moderating variable, and then created an interaction term of the standardized independent variable and moderating variable. Second, the dependent variable and the control variables were inserted into the equation. The independent variable and the moderating variable were then sequentially introduced. Third, we added the interaction term into the equation.

Based on the above analysis steps of moderating effect, the study analyzes moderating effects of green peer influence and “mianzi.” To simplify the contents presented in the table, only the analysis results of interaction items are presented here. Table 4 shows the hierarchical regression results. In Model 1 and Model 2, it can be seen that “mianzi” positively moderates the relationship between green self-identity and purchase intention of NEVs (β = 0.108, *p* < 0.05), but it does not moderate the relationship between personal norm and purchase intention (β = −0.006, *p* > 0.1). Therefore, H6b is supported, and H6a is rejected. In Model 3 and Model 4, green peer influence is observed to positively moderate the relationship between green self-identity and purchase intention of NEVs (β = 0.085, *p* < 0.1), but the coefficient of the moderating effect of green peer influence on the relationship between personal norm and purchase intention is not significant (β = −0.002, *p* > 0.1). Hence, H7b is supported, and H7a is rejected.

**TABLE 4:** Hierarchical regression results.

Dependent variables	Purchase Intention			
	M1	M2	M3	M4
**1. Control variables**				
Gender	0.093	0.086	0.166	0.169
Age	–0.094	–0.028	–0.124	–0.069
Education	0.103	0.108*	0.113*	0.109*
Occupation	0.036	0.036	0.038	0.032
Income	–0.013	0.019	–0.023	0.007
2. Independent variable Green self-identity	0.331***		0.270***	
Independent variable Personal norm		0.318***		0.238***
3. Moderating variable Mianzi	0.328***	0.368***		
Moderating variable Peer influence			0.340***	0.378***
4. Moderating effect				
Green self-identity* Mianzi	0.108**			
Personal norm* Mianzi		–0.006		
Green self-identity* Peer influence			0.085*	
Personal norm* Peer influence				–0.002
*R* ^2^	0.294	0.294	0.288	0.287
Adj. *R*^2^	0.275	0.275	0.269	0.268
*F*	15.267***	15.281***	14.837***	14.748***

*p < 0.1; **p < 0.05; ***p < 0.01.

**FIGURE 3:**
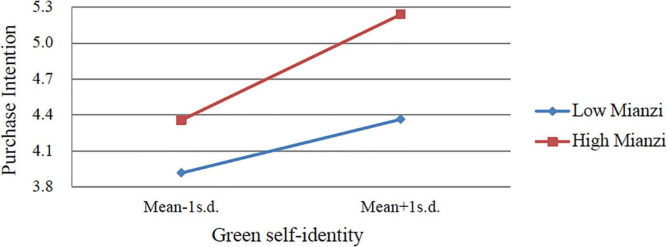
The moderating effect of “mianzi.”

**FIGURE 4:**
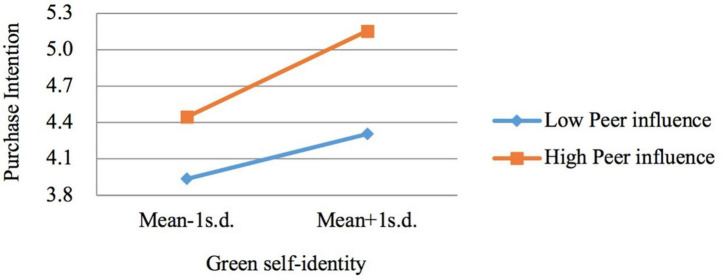
The moderating effect of green peer influence.

To explain the moderating effect intuitively, we provided a diagram that displays a moderating effect. We created high and low levels based on a standard deviation above and below the mean of moderating variable, and then we calculated the moderating effects (Dawson, 2014). As shown in Figures 3, 4, the relationship between green self-identity and purchase intention is significantly moderated by “mianzi” and green peer influence.

## Conclusion and policy implications

### Conclusion

Previous studies hold different views on the relationship between cultural factors and purchase intention of NEVs. Some studies believe that there is a direct relationship between the two, while others do not think so. In this study, cultural factors were used as moderating variables. According to the extended norm activation model, moral factors and cultural factors are combined to develop a research framework to explain the purchase of NEVs. That is to say, this study examines the influencing factors of purchase intention of NEVs and the moderating effects of “mianzi” and green peer influence. The key findings in this study are as follows.

First, awareness of consequence and ascription of responsibility are positively associated with the personal norm. This is aligned with the findings from He and Zhan (2018), suggesting awareness of consequence and ascription of responsibility are the predictors of the personal norm. When consumers are highly conscious of the potential effects of not using NEVs, personal norms are more inclined to protect the environment. Meanwhile, if consumers have a sense of responsibility and self-attribution for adverse environmental problems, they can easily develop environmental behavior for purchasing NEVs. Moreover, the personal norm has a positive correlation with purchase intention. This is consistent with the current research results that personal norm is positively connected to pro-environmental behavior (Song et al., 2019). When consumers are guided by personal norms, they have a moral obligation to buy eco-friendly products to minimize emissions.

Second, green self-identity plays a crucial role in creating personal norm and affects it positively. Green self-identity determines consumers’ intentions, consistent with previous studies (Bartels and Hoogendam, 2011; Neves and Oliveira, 2021). Individuals’ expectations of the appropriate behavior of their roles can strengthen their status in society. Consumers who deem themselves as green environmentalists are easy to see themselves as having a moral responsibility to carry out environmentally friendly behaviors (Camilla et al., 2017; Nguyen et al., 2016). In addition, it was achievable to improve the explanatory power of the extended model by adding green self-identity to the original NAM model.

Third, the relationship between green self-identity and purchasing intention is positively moderated by “mianzi.” Prior studies neglect to study cultural factors that are used as moderating variables (Chen et al., 2019). This finding of moderating effect adds to our understanding of the boundary condition of when green self-identity is vital for purchasing intention of NEVs. Chinese consumers may consider their social reputation when making purchase decisions because of the influence of Confucian culture (Guo and Lin, 2015). A consumer with high “mianzi” is concerned about their image in the public and is more inclined to purchase green products that can highlight their social status and public image. In addition, “mianzi” can be influenced by product attributes (Bao et al., 2003), such as green label and price. Green products have green features and can tag green image to purchasers. Therefore, consumers with green self-identity are more likely to buy NEVs when they are more inclined toward the concept of “mianzi.”

Fourth, green peer influence positively moderates the relationship between green self-identity and purchasing intention. This is aligned with the findings of Suki and Suki (2019), suggesting that peer influence can be used as a moderator. The powerful green peer influence will increase the effect of green self-identity on purchasing intention. The more consumers care about other people’s opinions, the more they intend to show their self-identity to others, as well to accelerate the conversion of green self-identity to purchasing intention.

Fifth, our study shows that “mianzi” and green peer influence do not moderate the connection between personal norm and purchase intention. The probable explanation is that the personal norm reflects the internal obligation and motivation to participate in pro-environment behavior in compliance with their moral conscience. This may make consumers less likely to be affected by external factors.

### Policy implications

This research has several management implications. The details are as follows.

First, personal norms play a critical role in encouraging the intention to purchase NEVs. The government needs to take measures to facilitate people’s moral norms to increase the purchase rate of NEVs and support the automotive industry’s growth. Publicity and education is a crucial way to popularize environmental knowledge to raise ecological awareness. A number of channels might be employed to promote environmental moral responsibility, including school education, media campaigns, and community education. The government should make use of the media to propagate the environmental performance of NEVs and purchase NEVs as a socially desirable behavior.

Second, the study reports that awareness of consequences, the ascription of responsibility, and green self-identity are essential antecedents of personal norms. The government should consider multiple media methods to publicize the severe environmental problems and detrimental effects of not using energy vehicles, such as haze, global warming, and the energy crisis. It should also be emphasized that people’s behavior and lifestyle are the major causes of environmental problems. Environmental non-governmental institutions and policymakers should conduct a range of publicity campaigns via various communication channels, such as creating public service advertising for ecological protection and organizing environmental protection publicity activities. Through the efforts of various institutions, people may gradually be aware of the adverse environmental impact of using traditional cars and feel responsible for improving the environmental benefits. Meanwhile, it is essential to enhance the green self-identity of each individual through the education system and inculcate environmental awareness of green consumption. The government should publicize that everyone can be a green consumer and that using NEVs is vital to one’s own self-concept. People should establish the identity of the green consumers and start from themselves to improve environmental performance with actual behavior.

Third, the study reports the importance of cultural factors in converting consumers’ green self-identity into the purchase intention of NEVs. The NEV is a technological innovation product with environmental protection function, but for consumers, the purchase of NEVs does not achieve a lot of symbolic meanings and social value. Enterprises should employ various information technology systems to publicize the environmental sustainability features of NEVs and help customers recognize the social value of NEVs. The advertisement design of NEVs should concentrate on the symbolic meanings and social prestige, so as to meet consumers’ need to improve their public recognition. Meanwhile, enterprises can provide some unique services and projects for NEV purchasers, so that consumers have a sense of superiority and “mianzi” to prove social status (Chen et al., 2019). Moreover, the government should play a significant role in publicizing the social status and reputation of purchasing NEVs, so that consumers obtain more social recognition and approval in social interaction. Besides, under the influence of collectivist culture, consumers tend to refer to others’ suggestions when making decisions and prefer to follow their role models. Similarly, when purchasing NEVs, consumers tend to refer to other people’s opinions. Thus, policymakers should issue detailed laws and regulations to encourage consumers to buy NEVs. Meanwhile, the government should try to set good examples to guide people to participate in positive pro-environment behavior (Blok et al., 2015).

### Limitations and future research

First, due to the difficulty in obtaining data on purchase behavior, this study collected data on purchase intention. This study uses purchase intention as a dependent variable. Although behavioral intention is a proxy variable of actual behavior (Hung et al., 2003), there is a chance that behavioral intention may not perfectly predict the actual behavior. Thus, further research should investigate the purchase behavior of NEVs.

Second, this article employs an extended norm activation model by adding psychological variables and cultural factors. There are other psychological variables and cultural factors that may influence the purchase of NEVs, such as environmental concerns, and Chinese typical family responsibility. To further enrich the relevant research on sustainable transport behavior, the future study may try to add other factors into the norm activation model to elaborate on the purchase behavior of NEVs.

Third, environmental problems have become a common concern in the world. Countries attach importance to sustainable development and ecological protection globally. However, different countries have different cultures and institutional contexts, different consumer behavior, and way of thinking. Therefore, there is a need to replicate this research in other countries to test the generalizability issues of the results of this study.

Fourth, the current research relies on self-reports. People are more likely to over-report their intentions related to the effect of social desirability, since purchasing new energy cars is typically considered as socially appropriate behavior. Therefore, future research can use longitudinal data to verify the research model.

## Data availability statement

The raw data supporting the conclusions of this article will be made available by the authors, without undue reservation.

## Ethics statement

Ethical review and approval was not required for the study on human participants in accordance with the local legislation and institutional requirements. Written informed consent from the participants was not required to participate in this study in accordance with the national legislation and the institutional requirements.

## Author contributions

HW and HZ conceived the study and wrote the first draft of the manuscript. RL was responsible for data collection. RB was responsible for revising and proofreading the manuscript. All authors contributed to the article and approved the submitted version.

## Footnotes

1
http://www.sojump.com/

## References

[B1] Arpita K. Shivendra P. (2017). Role of green self-identity and peer influence in fostering trust towards organic food retailers. *Int. J. Retail. Distrib.* 45 969–990. 10.1108/IJRDM-07-2016-0109

[B2] Bagozzi R. P. Yi Y. (1988). On the evaluation of structural equation models. *J. Acad. Market.* 16 74–94. 10.1007/BF02723327

[B3] Bao Y. Zhou K. Z. Su C. (2003). Face consciousness and risk aversion: Do they affect consumer decision-making? *Psychol. Market.* 20 733–755. 10.1002/mar.10094

[B4] Barbarossa C. De Pelsmacker P. (2016). Positive and negative antecedents of purchasingeco-friendly products: A comparison between green and non-green consumers. *J. Bus. Ethics* 134 229–247. 10.1007/s10551-014-2425-z

[B5] Bartels J. Hoogendam K. (2011). The role of social identity and attitudes toward sustainability brands in buying behaviors for organic products. *J. Brand. Manag*. 18 697–708. 10.1057/bm.2011.3

[B6] Bartels J. Reinders M. J. (2010). Social identification, social representations, and consumer innovativeness in an organic food context: A cross-national comparison. *Food Qual. Prefer.* 21 347–352. 10.1016/j.foodqual.2009.08.016

[B7] Bhattacharya C. B. Sen S. (2003). Consumer-company identification: A framework for understanding consumers’ relationships with companies. *J. Mark.* 67 76–88. 10.1509/jmkg.67.2.76.18609 11670861

[B8] Bjerkan K. Y. Nørbech T. E. Nordtømme M. E. (2016). Incentives for promoting battery electric vehicle (BEV) adoption in Norway. *Transp. Res. Part D Transp. Environ.* 43 169–180. 10.1016/j.trd.2015.12.002

[B9] Blok V. Wesselink R. Studynka O. Kemp R. (2015). Encouraging sustainability in the workplace: A survey on the pro-environmental behaviour of university employees. *J. Clean. Prod.* 106 55–67. 10.1016/j.jclepro.2014.07.063

[B10] Camilla B. De Patrick P. Ingrid M. (2017). Personal values, green self-identity and electric car adoption. *Ecol. Econ.* 140 190–200. 10.1016/j.ecolecon.2017.05.015

[B11] Chen K. Ren C. Gu R. Zhang P. (2019). Exploring purchase intentions of new energy vehicles: From the perspective of frugality and the concept of “mianzi”. *J. Clean. Prod.* 230 700–708. 10.1016/j.jclepro.2019.05.135

[B12] Chi Y. Y. Wang Y. Y. Xu J. H. (2021). Estimating the impact of the license plate quota policy for ICEVs on new energy vehicle adoption by using synthetic control method. *Energy Policy* 149:112022. 10.1016/j.enpol.2020.112022

[B13] Dawson J. F. (2014). Moderation in management research: What, why, when, and how. *J Bus Psychol.* 29 1–19. 10.1007/s10869-013-9308-7

[B14] De Groot J. I. Steg L. (2009). Morality and prosocial behavior: The role of awareness, responsibility, and norms in the norm activation model. *J. Soc. Psychol.* 149 425–449. 10.3200/SOCP.149.4.425-449 19702104

[B15] De Haan P. Mueller M. G. Peters A. (2006). Does the hybrid Toyota Prius lead to rebound effects: Analysis of size and number of cars previously owned by Swiss Prius buyers. *Ecol. Econ.* 58 592–605. 10.1016/j.ecolecon.2005.08.009

[B16] Dong X. Zhang B. Wang B. Wang Z. (2020). Urban households’ purchase intentions for pure electric vehicles under subsidy contexts in China: Do cost factors matter? *Transport. Res. A Pol.* 135 183–197. 10.1016/j.tra.2020.03.012

[B17] Energy Foundation [EF] (2020). *Synthesis report 2020 on China’s carbon neutrality—A new journey of China’s modernization: A new growth story from the “14th Five-Year Plan” to carbon neutrality.* Beijing: Energy Foundation.

[B18] Faiers A. Cook M. Neame C. (2007). Towards a contemporary approach for understanding consumer behaviour in the context of domestic energy use. *Energy Policy* 35 4381–4390. 10.1016/j.enpol.2007.01.003

[B19] Fornell C. Larcker D. F. (1981). Evaluating structural equation models with unobservable variables and measurement error. *J. Mark. Res*. 18 39–50. 10.1177/002224378101800104

[B20] Graham-Rowe E. Gardner B. Abraham C. Skippon S. Dittmar H. Hutchins R. (2012). Mainstream consumers driving plug-in battery-electric and plug-in hybrid electric cars: A qualitative analysis of responses and evaluations. *Transport. Res. A Pol*. 46 140–153. 10.1016/j.tra.2011.09.008

[B21] Guo X. L. Lin D. R. (2015). A review of Chinese consumer face awareness and consumer behavior. *Foreign Econ. Manag*. 37 63–71.

[B22] Han H. (2014). The norm activation model and theory-broadening: Individuals’ decision-making on environmentally-responsible convention attendance. *J. Environ. Psychol.* 40 462–471. 10.1016/j.jenvp.2014.10.006

[B23] Harland P. Staats H. Wilke H. A. (2007). Situational and personality factors as direct or personal norm mediated predictors of pro-environmental behavior: Questions derived from norm-activation theory. *Basic Appl. Soc*. *Psychol.* 29 323–334. 10.1080/01973530701665058

[B24] He J. X. Li J. Y. Zhao D. Q. Chen X. (2022). Does oil price affect corporate innovation? Evidence from new energy vehicle enterprises in China. *Renew. Sust. Energ. Rev*. 156 111964. 10.1016/j.rser.2021.111964

[B25] He W. Y. He R. (2015). Empirical analysis on the influencing factors of public market diffusion of new energy vehicles. *J. Dalian Univ. Technol.* 36 28–33.

[B26] He X. Zhan W. (2018). How to activate moral norm to adopt electric vehicles in China? An empirical study based on extended norm activation theory. *J. Clean. Prod.* 172 3546–3556. 10.1016/j.jclepro.2017.05.088

[B27] He Z. X. Zhou Y. Q. Wang J. M. Li C. F. Wang M. L. Li W. B. (2020). The impact of motivation, intention, and contextual factors on green purchasing behavior: New energy vehicles as an example. *Bus. Strateg. Environ*. 30 1249–1269. 10.1002/bse.2682

[B28] Hofmann J. Guan D. Chalvatzis K. Huo H. (2016). Assessment of electrical vehicles as a successful driver for reducing CO2 emissions in China. *Appl. Energy.* 184 995–1003. 10.1016/j.apenergy.2016.06.042

[B29] Hopper J. R. Nielsen J. M. (1991). Recycling as altruistic behavior: Normative and behavioral strategies to expand participation in a community recycling program. *Environ. Behav.* 23 195–220. 10.1177/0013916591232004

[B30] Huang X. Ge J. (2019). Electric vehicle development in Beijing: An analysis of consumer purchase intention. *J. Clean. Prod*. 216 361–372. 10.1016/j.jclepro.2019.01.231

[B31] Hung S.-Y. Ku C.-Y. Chang C.-M. (2003). Critical factors of WAP services adoption: An empirical study. *Electr. Commer. R A.* 2 42–60. 10.1016/S1567-4223(03)00008-5

[B32] Jiang C. F. (2009). *Face and consumption.* Beijing: Social Sciences Academic Press.

[B33] Kang M. J. Park H. (2011). Impact of experience on government policy toward acceptance of hydrogen fuel cell vehicles in Korea. *Energy Policy* 39 3465–3475. 10.1016/j.enpol.2011.03.045

[B34] Kelly S. F. Rachel M. D. Winnifred R. L. (2008). Theory of planned behaviour, identity and intentions to engage in environmental activism. *J. Environ. Psychol*. 28 318–326. 10.1016/j.jenvp.2008.03.003

[B35] Kim M. K. Oh J. Park J. H. Joo C. (2018). Perceived value and adoption intention for electric vehicles in Korea: Moderating effects of environmental traits and government supports. *Energy* 159 799–809. 10.1016/j.energy.2018.06.064

[B36] Lao K. F. Wang L. L. (2015). The influence of Chinese traditional cultural values on environmental behavior based on green product purchasing behavior of consumers. *J. Shanghai Univ. Finance Econ.* 17 64–74.

[B37] Lee K. (2008). Opportunities for green marketing: Young consumers. *Mark. Intell. Plan.* 26 573–586. 10.1108/02634500810902839 32057434

[B38] Lee K. (2009). Gender differences in Hong Kong adolescent consumers’ green purchasing behavior. *J. Consum. Mark.* 26 87–96. 10.1108/07363760910940456

[B39] Li L. Wang Z. Chen L. Wang Z. (2020). Consumer preferences for battery electric vehicles: A choice experimental survey in China. *Transport. Res. Part D*. 78 102185. 10.1016/j.trd.2019.11.014

[B40] Lin B. Q. Shi L. (2022). Identify and bridge the intention-behavior gap in new energy vehicles consumption: Based on a new measurement method. *Sustain. Prod. Consump*. 31 432–447. 10.1016/j.spc.2022.03.015

[B41] Linn J. McConnell V. (2019). Interaction between federal and state policies for reducing vehicle emissions. *Energy Policy* 126 507–517. 10.1016/j.enpol.2018.10.052 31883240

[B42] Liu C. Liu Y. Zhang D. Y. Xie C. P. (2022). The capital market responses to new energy vehicle (NEV) subsidies: An event study on China. *Energ. Econ.* 105 105677. 10.1016/j.eneco.2021.105677

[B43] Liu W. L. Zeng L. L. Wang Q. W. (2021). Psychological distance toward air pollution and purchase intention for new energy vehicles: An investigation in China. *Front. Psychol*. 12:569115. 10.3389/fpsyg.2021.569115 33868068 PMC8046919

[B44] Lopes M. M. Moura F. Martinez L. M. (2014). A rule-based approach for determining the plausible universe of electric vehicle buyers in the Lisbon Metropolitan Area. *Transport. Res. A Pol.* 59 22–36. 10.1016/j.tra.2013.09.009

[B45] Malhotra N. K. Kim S. S. Patil A. (2006). Common method variance in is research: A comparison of alternative approaches and a reanalysis of past research. *Manage Sci*. 52 1865–1883. 10.1287/mnsc.1060.0597 19642375

[B46] Miao Z. Baležentis T. Shao S. Chang D. (2019). Energy use, industrial soot and vehicle exhaust pollution—China’s regional air pollution recognition, performance decomposition and governance. *Energy Econ*. 83 501–514. 10.1016/j.eneco.2019.07.002

[B47] Nekmahmud M. Ramkissoon H. Fekete-Farkas M. (2022). Green purchase and sustainable consumption: A comparative study between European and non-European tourists. *Tour. Manag. Perspect*. 43:100980. 10.1016/j.tmp.2022.100980

[B48] Neves J. Oliveira T. (2021). Understanding energy-efficient heating appliance behavior change: The moderating impact of the green self-identity. *Energy* 225:120169. 10.1016/j.energy.2021.120169

[B49] Nguyen T. N. Lobo A. Greenland S. (2016). Pro-environmental purchase behaviour: The role of consumers’ biospheric values. *J. Retail. Consum. Serv.* 33 98–108. 10.1016/j.jretconser.2016.08.010

[B50] Nordlund A. M. Garvill J. (2003). Effects of values, problem awareness, and personal norm on willingness to reduce personal car use. *J. Environ. Psychol*. 23 339–347. 10.1016/S0272-4944(03)00037-9

[B51] Ozaki R. Sevastyanova K. (2011). Going hybrid: An analysis of consumer purchase motivations. *Energy Policy*. 39 2217–2227. 10.1016/j.enpol.2010.04.024

[B52] Paulraj A. Lado A. A. Chen I. J. (2008). Inter-organizational communication as a relational competency: Antecedents and performance outcomes in collaborative buyeresupplier relationships. *J. Oper. Manag.* 26 45–64. 10.1016/j.jom.2007.04.001

[B53] Peters A. De Haan P. Scholz R. W. (2015). Understanding car-buying behavior: Psychological determinants of energy efficiency and practical implications. *Int. J. Sustain Transp.* 9 59–72. 10.1080/15568318.2012.732672

[B54] Podsakoff P. M. MacKenzie S. B. Lee J.-Y. Podsakoff N. P. (2003). Common method biases in behavioral research: A critical review of the literature and recommended remedies. *J. Appl. Psychol.* 88 879–903. 10.1037/0021-9010.88.5.879 14516251

[B55] Qin S. F. Xiong Y. Q. (2022). Innovation strategies of Chinese new energy vehicle enterprises under the influence of non-financial policies: Effects, mechanisms and implications. *Energy Policy*. 164 112946. 10.1016/j.enpol.2022.112946

[B56] Schwartz S. H. (1973). Normative explanations of helping behavior: A critique, proposal, and empirical test. *J. Exp. Soc. Psychol.* 9 349–364. 10.1016/0022-1031(73)90071-1

[B57] Schwartz S. H. (1977). Normative influences on altruism. *Adv. Exp. Soc. Psychol.* 10 221–279. 10.1016/S0065-2601(08)60358-5

[B58] Shi H. Fan J. Zhao D. (2017). Predicting household PM2.5-reduction behavior in Chinese urban areas: An integrative model of theory of planned behavior and norm activation theory. *J. Clean. Prod.* 145 64–73. 10.1016/j.jclepro.2016.12.169

[B59] Sirgy M. J. (1986). *Self-congruity: Toward a theory of personality and cybernetics.* Westport, CT: Greenwood Publishing Group.

[B60] Smith J. R. McSweeney A. (2007). Charitable giving: The effectiveness of a revised theory of planned behaviour model in predicting donating intentions and behaviour. *J. Community Appl. Soc. Psychol.* 17 363–386. 10.1002/casp.906

[B61] Song Y. Zhao C. Zhang M. (2019). Does haze pollution promote the consumption of energy-saving appliances in China? An empirical study based on norm activation model. *Resour. Conserv. Recy*. 145 220–229. 10.1016/j.resconrec.2019.02.041

[B62] Steg L. Dreijerink L. Abrahamse W. (2005). Factors influencing the acceptability of energy policies: A test of VBN theory. *J. Environ. Psychol.* 25 415–425. 10.1016/j.jenvp.2005.08.003

[B63] Su D. Gu Y. Du Q. Zhou W. Huang Y. (2020). Factors affecting user satisfaction with new energy vehicles: A field survey in Shanghai and Nanjing. *J. Environ. Manage.* 270 110857. 10.1016/j.jenvman.2020.110857 32721306

[B64] Suki N. M. Suki N. M. (2019). Examination of peer influence as a moderator and predictor in explaining green purchase behaviour in a developing country. *J. Clean. Prod.* 228 833–844. 10.1016/j.jclepro.2019.04.218

[B65] Tan R. Tang D. Lin B. (2018). Policy impact of new energy vehicles promotion on air quality in Chinese cities. *Energy Policy* 118 33–40. 10.1016/j.enpol.2018.03.018

[B66] Tsarenko Y. Ferraro C. Sands S. Mclead C. (2013). Environmentally conscious consumption: The role of retailers and peers as external influences. *J. Retail. Consum. Serv.* 20 302–310. 10.1016/j.jretconser.2013.01.006

[B67] van der Werff E. Steg L. Keizer K. (2013). The value of environmental self-identity: The relationship between biospheric values, environmental self-identity and environmental preferences, intentions and behaviour. *J. Environ. Psychol.* 34 55–63. 10.1016/j.jenvp.2012.12.006

[B68] Vignoles V. L. Regalia C. Manzi C. Golledge J. Scabini E. (2006). Beyond self-esteem: Influence of multiple motives on identity construction. *J. Pers. Soc. Psychol*. 90 308–333. 10.1037/0022-3514.90.2.308 16536653

[B69] Wang J. M. (2013). The effect of resource conservation consciousness on resource saving behavior: An interactive and moderating effect model in the Chinese cultural context. *Manag. World* 8 77–100.

[B70] Wang L. Fu Z.-L. Guo W. Liang R.-Y. Shao H.-Y. (2020). What influences sales market of new energy vehicles in China? Empirical study based on survey of consumers’ purchase reasons. *Energy Policy* 142 111484. 10.1016/j.enpol.2020.111484

[B71] Wang R. Zhao X. Y. Wang W. J. Jiang L. (2021). What factors affect the public acceptance of new energy vehicles in underdeveloped regions? A case study of Gansu Province, China. *J. Clean. Prod.* 318 128432. 10.1016/j.jclepro.2021.128432

[B72] Wang Y. H. Wang Q. (2013). Factors affecting Beijing residents’ buying behavior of new energy vehicle: An integration of technology acceptance model and theory of planned behavior. *Chin. J. Manag. Sci.* 21 691–698.

[B73] Wang Z. H. Guo D. X. Wang X. M. Zhang B. Wang B. (2018). How does information publicity influence residents’ behaviour intentions around e-waste recycling? *Resour. Conserv. Recycl.* 133 1–9. 10.1016/j.resconrec.2018.01.014

[B74] Wang Z. Dong X. (2016). Determinants and policy implications of residents’ new energy vehicle purchases: The evidence from China. *Nat. Hazards* 82 155–173. 10.1007/s11069-016-2185-4

[B75] Wittenberg I. Blöbaum A. Matthies E. (2018). Environmental motivations for energy use in PV households: Proposal of a modified norm activation model for the specific context of PV households. *J. Environ. Psychol*. 55 110–120. 10.1016/j.jenvp.2018.01.002

[B76] Wu J. X. Wu H. C. Hsieh C. M. Ramkissoon H. (2022). Face consciousness, personal norms, and environmentally responsible behavior of Chinese tourists: Evidence from a lake tourism site. *J. Hosp. Tour. Manag*. 50 148–158. 10.1016/j.jhtm.2022.01.010

[B77] Xu G. Wang S. Li J. Zhao D. (2020). Moving towards sustainable purchase behavior: Examining the determinants of consumers’intentions to adopt electric vehicles. *Environ. Sci. Pollut. Res*. 27 22535–22546. 10.1007/s11356-020-08835-9 32319053

[B78] Yadav R. Pathak G. S. (2016a). Intention to purchase organic food among young consumers: Evidences from a developing nation. *Appetite* 96 122–128. 10.1016/j.appet.2015.09.017 26386300

[B79] Yadav R. Pathak G. S. (2016b). Young consumers’ intention towards buying green products in a developing nation: Extending the theory of planned behavior. *J. Clean. Prod.* 135 732–739. 10.1016/j.jclepro.2016.06.120

[B80] Yu F. Wang L. Li X. (2020). The effects of government subsidies on new energy vehicle enterprises: The moderating role of intelligent transformation. *Energy Policy* 141 111463. 10.1016/j.enpol.2020.111463

[B81] Zhang X. A. Cao Q. Grigoriou N. (2011). Consciousness of social face: Development and validation of a scale measuring desire to gain face versus fear of losing face. *J. Soc. Psychol.* 151 129–149. 10.1080/00224540903366669 21476458

[B82] Zhang X. Wang K. Hao Y. Fan J. L. Wei Y. M. (2013). The impact of government policy on preference for NEVs: The evidence from China. *Energy Policy* 61 382–393. 10.1016/j.enpol.2013.06.114

[B83] Zhang Y. Peng H. Liu Z. Tan W. (2015). Direct energy rebound effect for road passenger transport in China: A dynamic panel quantile regression approach. *Energy Policy* 87 303–313. 10.1016/j.enpol.2015.09.022

[B84] Zhang Y. Wang Z. Zhou G. (2013). Antecedents of employee electricity saving behavior in organizations: An empirical study based on norm activation model. *Energy Policy* 62 1120–1127. 10.1016/j.enpol.2013.07.036

[B85] Zhao D. Ji S. F. Wang H. P. Jiang L. W. (2021). How do government subsidies promote new energy vehicle diffusion in the complex network context? A three-stage evolutionary game model. *Energy* 230:120899. 10.1016/j.energy.2021.120899 PMC9761936 36568911

